# Revisiting prognostic factors in glioma with leptomeningeal metastases: a comprehensive analysis of clinical and molecular factors and treatment modalities

**DOI:** 10.1007/s11060-022-04233-y

**Published:** 2023-02-25

**Authors:** Yae Won Park, Kyunghwa Han, Sooyon Kim, Hyuk Kwon, Sung Soo Ahn, Ju Hyung Moon, Eui Hyun Kim, Jinna Kim, Seok-Gu Kang, Jong Hee Chang, Se Hoon Kim, Seung-Koo Lee

**Affiliations:** 1 Department of Radiology and Research Institute of Radiological Science and Center for Clinical Imaging Data Science, Yonsei University College of Medicine, 50-1 Yonsei-Ro, Seodaemun-Gu, Seoul, 120-752 Korea; 2 Department of Statistics and Data Science, Yonsei University, Seoul, Korea; 3 Sea Salvage & Rescue Unit, Naval Special Warfare Flotilla, Gyeryong, Korea; 4 Department of Neurosurgery, Yonsei University College of Medicine, Seoul, Korea; 5 Department of Pathology, Yonsei University College of Medicine, Seoul, Korea

**Keywords:** Glioma, Isocitrate dehydrogenase, Magnetic resonance imaging, Leptomeningeal metastases, Survival

## Abstract

**Purpose:**

To comprehensively investigate prognostic factors, including clinical and molecular factors and treatment modalities, in adult glioma patients with leptomeningeal metastases (LM).

**Methods:**

Total 226 patients with LM (from 2001 to 2021 among 1495 grade 2 to 4 glioma patients, 88.5% of LM patients being IDH-wildtype) with complete information on IDH mutation, 1p/19q codeletion, and MGMT promoter methylation status were enrolled. Predictors of overall survival (OS) of entire patients were determined by time-dependent Cox analysis, including clinical, molecular, and treatment data. Subgroup analyses were performed for patients with LM at initial diagnosis and LM diagnosed at recurrence (herein, initial and recurrent LM). Identical analyses were performed in IDH-wildtype glioblastoma patients.

**Results:**

Median OS was 17.0 (IQR 9.7–67.1) months, with shorter median OS in initial LM than recurrent LM patients (12.2 vs 20.6 months, *P* < 0.001). In entire patients, chemotherapy and antiangiogenic therapy were predictors of longer OS, while male sex and initial LM were predictors of shorter OS. In initial LM, higher KPS, chemotherapy, and antiangiogenic therapy were predictors of longer OS, while male sex was a predictor of shorter OS. In recurrent LM, chemotherapy and longer interval between initial glioma and LM diagnoses were predictors of longer OS, while male sex was a predictor of shorter OS. A similar trend was observed in IDH-wildtype glioblastoma.

**Conclusion:**

Active chemotherapy and antiangiogenic therapy demonstrated survival benefit in glioma patients with LM. There is consistent female survival advantage, whereas longer interval between initial glioma diagnosis and LM development suggests longer OS in recurrent LM.

**Supplementary Information:**

The online version contains supplementary material available at 10.1007/s11060-022-04233-y.

## Introduction

Leptomeningeal metastases (LM), which indicate tumor cell invasion into leptomeninges via either direct invasion or cerebrospinal fluid (CSF), is formerly considered a devastating complication in glioma patients, with 75% of patients dying within 18 months of diagnosis in previous reports [1]. LM has been previously considered a rare and terminal condition, and active treatment has not been always pursued. However, the overall incidence of LM has been gradually increasing, which may be attributed to advances in glioma treatment with increasing overall survival (OS) [1] as well as implementation of diagnostic modality with high sensitivity. The reported incidence of LM on autopsy is approximately 21.2% [2], and our study with CSF-sensitive imaging revealed an incidence up to 16.2%, [3] suggesting LM is an underdiagnosed condition. Moreover, LM may no longer be an end-stage complication in diffuse gliomas; approximately half of the patients with LM were identified during initial glioma diagnosis in our previous study [3].

The high incidence of LM suggests that active treatment should be considered if treatment is feasible and effective. Multiple treatment modalities such as chemotherapy and radiation therapy have been reported to increase OS of glioma patients with LM [1, 4]. In contrast, antiangiogenic treatment has shown discrepant results; some studies reported a survival benefit [5, 6], while others have not [7–10]. However, previous studies were performed with small datasets, some with less than 10 patients with LM, which questions the validity of these results [1]. Moreover, studies mostly focused only on glioblastoma and did not examine non-glioblastoma patients who can also develop LM, albeit the lower risk [1]. Thus, there is no standardized treatment guideline in glioma patients with LM.

A comprehensive analysis evaluating the effect of clinical, imaging, and molecular data on survival of patients with LM is lacking. Important molecular markers in the World Health Organization (WHO) classification such as isocitrate dehydrogenase (IDH) mutation and 1p/19q codeletion [11], as well as O^6^-methylguanine-DNA methyltransferase (MGMT) promoter methylation were not thoroughly reflected in previous studies [1]. Thus, it is unknown whether such factors affect OS of patients with LM in this molecular era. A thorough investigation to determine possible prognostic factors in patients with LM may inform clinicians who manage patients with a high risk of deterioration.

Therefore, this study aimed to comprehensively investigate prognostic factors such as clinical and molecular factors and treatment modalities in adult patients with glioma with LM.

## Methods

### Study design and ethical approval

Requirement for patient consent was waived in this retrospective study by the Institutional Review Board of the Severance Hospital (Approval Number 4-2021-1638).

## Patient enrollment

**Fig. 1:**
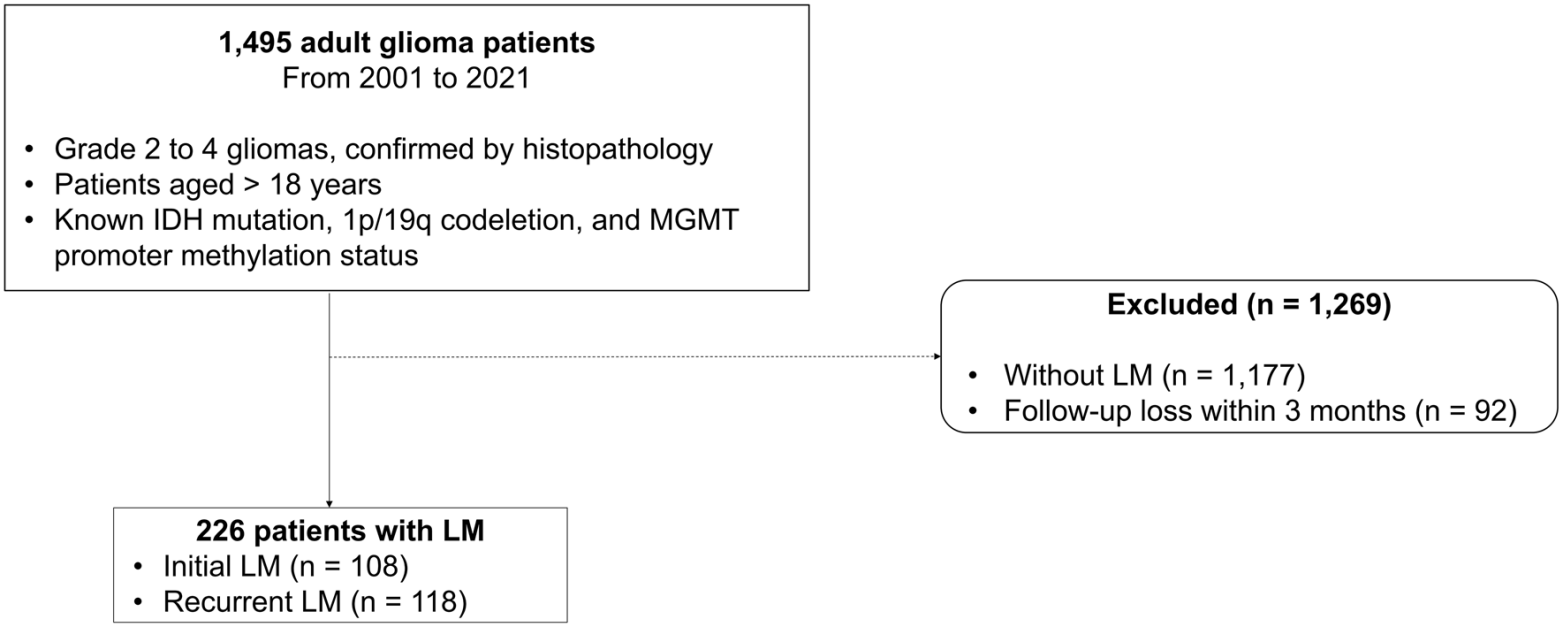
Flowchart for patient selection. *LM* leptomeningeal metastases

Between March 2001 and October 2021, 1495 adult patients with glioma from our institution were enrolled. Inclusion criteria were: (1) grade 2 to 4 gliomas confirmed on histopathology, (2) known IDH mutation, 1p/19q codeletion, and MGMT promoter methylation status, and (3) aged > 18 years. Exclusion criteria were: (1) without LM diagnosis on MRI or CSF examinations (n = 1177), and (2) follow-up loss within 3 months (n = 92). Total 226 patients were enrolled. Figure 1 shows the flow chart of glioma patients with LM.

### Molecular classification

All surgical tissues were diagnosed according to the 2016 WHO classification [12]. IDH mutation status was assessed by direct sequencing or immunohistochemistry using the R132H mouse monoclonal antibody (clone H09 L, Dianova; 1:80 dilution). For cases that predated the routine use of IDH mutational testing, formalin-fixed, paraffin-embedded blocks were reexamined for both IDH1 and IDH2 mutation statuses using a targeted next-generation sequencing panel (Trusight Tumor 170, Illumina) [13]. Patients younger than 55 years with negative immunostaining for IDH1 mutation were followed by sequencing for mutations in IDH1 and IDH2 according to the European Association for Neuro-Oncology guideline [14]. Fluorescent in situ hybridization analysis was performed to detect 1p/19q codeletion. MGMT promoter methylation status was determined by methylation-specific polymerase chain reaction until 2016 [15]; after that, pyrosequencing was used to quantify MGMT methylation levels using a PyroMark Q24 system (Qiagen). The presence of histone H3 K27M mutant protein was evaluated by immunohistochemistry analysis using polyclonal antibodies to detect the histone H3.3 tail in appropriate clinical and pathological settings, as previously recommended. [16]

For copy number analysis, epidermal growth factor receptor (EGFR) genes with ≥ two-fold change relative to the average level were considered to have undergone amplification [17]. Telomerase reverse transcriptase promoter (TERTp) mutation was determined using a pyrosequencing assay, and C228T and C250T mutations were also analyzed. EGFR amplification and TERTp mutation statuses were noted in subsets of 196 (86.7%) and 159 (70.4%) patients, respectively.

### MRI protocol

Brain MRI, including T1-weighted, T2-weighted, pre- and postcontrast FLAIR, [18, 19] and postcontrast 3D T1-weighted images, was performed using a 3-T unit (Achieva or Ingenia; Philips). Spine MRI, including T1-weighted, T2-weighted, and postcontrast T1-weighted images, were acquired using a 1.5-T unit (Achieva dStream; Philips). Detailed MRI protocol is in Supplementary Material S1.

### Data collection

Data on patient age at initial glioma diagnosis, sex, date of initial glioma diagnosis, date of LM diagnosis, MRI findings, CSF results, Karnofsky performance status (KPS) at initial glioma diagnosis, KPS at tumor recurrence, initial and subsequent treatments before and after recurrence, and date of death or last follow-up were collected.

Location of the glioma was recorded [20]. Resection extent was categorized (gross tumor removal, subtotal [tumor removal, ≥ 75% but < 100%), partial [tumor removal, < 75%) or biopsy] based on postoperative imaging as previously described. [21, 22]

OS was defined as the time from glioma diagnosis until death or last follow-up.

### Diagnosis of LM

LM was diagnosed in patients whose brain or spine MRI showed LM or who had positive CSF cytology results. MRI criteria for LM diagnosis was described in a previous study [23]. Disseminated and subependymal LM were separately recorded according to the previous criteria [7]. Disseminated LM was defined as leptomeningeal or nerve root enhancement, whereas subependymal LM was defined as a subependymal or ependymal enhancement. Details of LM diagnosis are in Supplementary Material S2.

### Statistical analysis

In all patients with LM, time-dependent Cox analysis was performed [24]. LM diagnosis, KPS at initial glioma diagnosis and recurrence, and initial and subsequent treatments before and after recurrence are time-dependent factors and was defined as such rather than as fixed covariates [24]. A time-dependent variable is incorporated into the analysis as a single value according to the repeated observation intervals [24]. Variables of interest with in the univariable Cox analysis (*P* < 0.05) were included in the multivariable Cox models using backward elimination according to the likelihood ratio.

Subgroup analyses were performed for patients with LM at initial diagnosis (herein, initial LM) and LM at recurrence (herein, recurrent LM) using univariable and multivariable Cox analyses. The proportional hazards assumption was met in these models on tests based on Schoenfeld residuals.

Identical analyses were performed in IDH-wildtype glioblastoma patients with LM; time-dependent Cox analysis was performed in all IDH-wildtype glioblastoma patients, followed by subgroup analyses in initial LM and recurrent LM patients.

In the time-dependent Cox analysis, survival rates were determined using the Simon-Makuch method [25]. In the Cox analyses for subgroups, survival rates were determined using the unadjusted and adjusted Kaplan–Meier methods, and curves were compared using the log-rank test. Statistical analyses were performed using R studio (version 1.1.456). Statistical significance was set at *P* values < 0.05. A biostatistician (with 11 years of experience) was consulted.

## Results

### Patient characteristics

This study included 226 glioma patients with LM (mean age: 56.1 ± 14.2 years, 82 females and 144 males), with a median follow-up period of 18.8 (interquartile range [IQR] 10.1–28.7) months. There were 3 patients with oligodendroglioma (1.3%; one with grade 2 and two with grade 3); 11 patients with IDH-mutant astrocytoma (4.9%; seven with grade 2, one with grade 3, and three with grade 4); 200 patients with IDH-wildtype astrocytoma (88.5%; two with grade 2, 19 grade 3, and 179 grade 4), and 12 patients with H3 K27M-altered diffuse midline glioma (6.3%). Seventy-one patients (31.4%) had MGMT promoter methylation. Based on the date of LM diagnosis, 108 (47.8%) and 118 (52.8%) patients had initial and recurrent LMs, respectively. The median interval between initial glioma and LM diagnoses was 11.4 (IQR 6.6–20.8) months in recurrent LM patients.

Among patients with LM, 222 of 226 patients (98.2%) who underwent brain MRI was positive on brain MRI, and 69 of 101 patients (68.3%) who underwent spine MRI was positive on spine MRI, while 12 of 61 patients (19.7%) who underwent CSF cytology was positive. According to the MRI criteria, 181 (80.1%) and 45 (19.9%) patients had disseminated and subependymal LMs, respectively. The median OS was 17.0 (IQR 9.7–67.1) months, and 151 patients (66.8%) died. The median OS significantly differed according to the molecular subgroups of glioma (log-rank *P* < 0.001). The Kaplan–Meier curve of OS according to the molecular subgroups is in Supplementary Fig. 1.

### Comparison of patients with LM at initial diagnosis and recurrence

Characteristics of patients with initial and recurrent LM are summarized in Table 1. The age at initial diagnosis was higher in initial LM than recurrent LM patients (58.8 vs. 53.6, *P* = 0.005). Patients with initial LM had a significantly higher proportion of grade 4 gliomas than those with recurrent LM (94.4% vs. 77.1%, *P* = 0.001).

**Table 1:** Characteristics of glioma patients with LM

Characteristics	Patients with LM (n = 226)	Initial LM (n = 108)	Recurrent LM (n = 118)	*P* *
Age at initial glioma diagnosis (years)	56.1 ± 14.2	58.8 ± 12.7	53.6 ± 15.0	0.005
Interval between initial glioma and LM diagnoses (months)	–	0 (0)	11.4 (6.6–20.8)	–
Sex, female	82 (36.3)	35 (32.4)	47 (39.8)	0.246
Histological grade				0.001
Grade 2	10 (4.4)	1 (0.9)	9 (7.6)	
Grade 3	23 (10.2)	5 (4.6)	18 (15.3)	
Grade 4	193 (85.4)	102 (94.4)	91 (77.1)	
Molecular subgroup				0.158
IDH mutant and 1p/19 codeletion	3 (1.3)	1 (0.9)	2 (1.7)	
IDH mutant and no 1p/19 codeletion	11 (4.9)	3 (2.8)	8 (6.8)	
IDH wildtype	200 (88.5)	101 (93.5)	99 (83.9)	
H3 K27M alteration	12 (6.3)	3 (2.8)	9 (7.6)	
Other molecular markers				
MGMT methylation	71 (31.4)	31 (28.7)	40 (33.9)	0.401
EGFR amplification^a^	48 (24.4)	25 (28.1)	23 (21.5)	0.286
TERTp mutation^b^	58 (36.5)	34 (42.5)	34 (43.0)	0.945
Nonlobar location	70 (31.0)	35 (32.4)	35 (29.7)	0.656
Infratentorial location	12 (5.3)	3 (2.8)	9 (7.6)	0.104
LM diagnosis, positive/tested (%)				
Brain MRI	222/226 (98.2)	108/108 (100)	114/118 (96.6)	0.054
Spine MRI	69/101 (68.3)	35/55 (63.6)	34/46 (73.9)	0.271
CSF cytology	12/61 (19.7)	4/24 (16.7)	8/37 (21.6)	0.637
Type of LM				0.243
Disseminated LM	181 (80.1)	90 (83.3)	91 (77.1)	
Subependymal LM	45 (19.9)	18 (16.7)	27 (22.9)	
KPS at initial glioma diagnosis	80 (70–80)	80 (70–80)	70 (70–80)	0.398
KPS at recurrence^c^	60 (50–70)	60 (40–60)	60 (50–70)	0.015
Gross total resection	106 (46.9)	43 (39.8)	63 (53.4)	0.041
Treatment before recurrence				
Chemotherapy	220 (97.3)	104 (96.3)	117 (99.2)	0.145
Radiation therapy	223 (98.7)	105 (97.2)	118 (100)	0.068
VP shunt	12 (5.3)	24 (22.2)	16 (13.6)	0.088
Antiangiogenic therapy	39 (1.7)	0 (0)	12 (10.2)	< 0.001
Treatment after recurrence				
Chemotherapy	18 (8.0)	4 (3.7)	14 (11.9)	0.024
Radiation therapy	22 (9.7)	5 (4.6)	17 (14.4)	0.013
VP shunt	20 (8.8)	3 (2.8)	27 (14.4)	0.002
Antiangiogenic therapy	78 (34.5)	21 (19.4)	57 (48.3)	< 0.001
Experimental therapy^d^	4 (1.8)	1 (0.9)	3 (2.5)	0.357
Death	151 (66.8)	67 (62.0)	84 (71.2)	0.145
OS after glioma diagnosis (months)	17.0 (9.7–37.1)	12.2 (6.9–23.2)	20.6 (12.4–40.3)	< 0.001

Data are presented as counts (%), mean ± standard deviation, or median (interquartile range)

*EGFR* epidermal growth factor receptor, *IDH* isocitrate dehydrogenase, *IQR* interquartile range, *LM* leptomeningeal metastases, *MGMT* O^6^-methylguanine-methyltransferase, *OS* overall survival, *TERTp* telomerase reverse transcriptase promoter, *VP* ventriculoperitoneal

*Comparison between patients with initial LM and recurrent LM

^a^Data of 196 patients with available information

^b^Data of 159 patients with available information

^c^Data of 169 patients with available information

^d^Three patients received lenvatinib plus pembrolizumab treatment, while one patient received belvarafenib treatment

Among patients with initial LM, 104 (96.3%) and 105 (97.2%) patients received chemotherapy and radiation therapy, respectively, at initial diagnosis. Among patients with initial LM who received radiation therapy, 15 (13.9%), 11 (10.2%), and 79 (73.1%) patients received craniospinal irradiation, whole ventricular radiation therapy, and localized radiation therapy, respectively. Antiangiogenic therapy was not performed at initial diagnosis. When recurrence was diagnosed, repeated radiation therapy was administered to five (4.6%) patients, with one (0.9%) receiving whole ventricular radiation therapy. Antiangiogenic therapy was administered to 21 (19.4%) patients with initial LM when recurrence was diagnosed, while one (0.9%) patient received experimental therapy with lenvatinib plus pembrolizumab on recurrence.

Among patients with recurrent LM, 14 (11.9%) and 17 (14.4%) patients received chemotherapy and radiation therapy, respectively, at LM diagnosis at recurrence. Among patients with recurrent LM who received radiation therapy on LM diagnosis, six (35.3%) and 11 (64.7%) patients received whole ventricular radiation therapy and localized radiation therapy, respectively. Antiangiogenic therapy was administered to 57 (48.3%) patients when recurrent LM was diagnosed. Two (1.7%) patients received experimental therapy with lenvatinib plus pembrolizumab, while one (0.8%) patient received belvarafenib treatment.

The median OS was significantly shorter in patients with initial LM than in those with recurrent LM (12.2 [IQR 6.9–23.2] vs 20.6 [IQR 12.4–40.3] months; log-rank test *P* < 0.001).

### Predictors of OS in all patients with LM

**Fig. 2:**
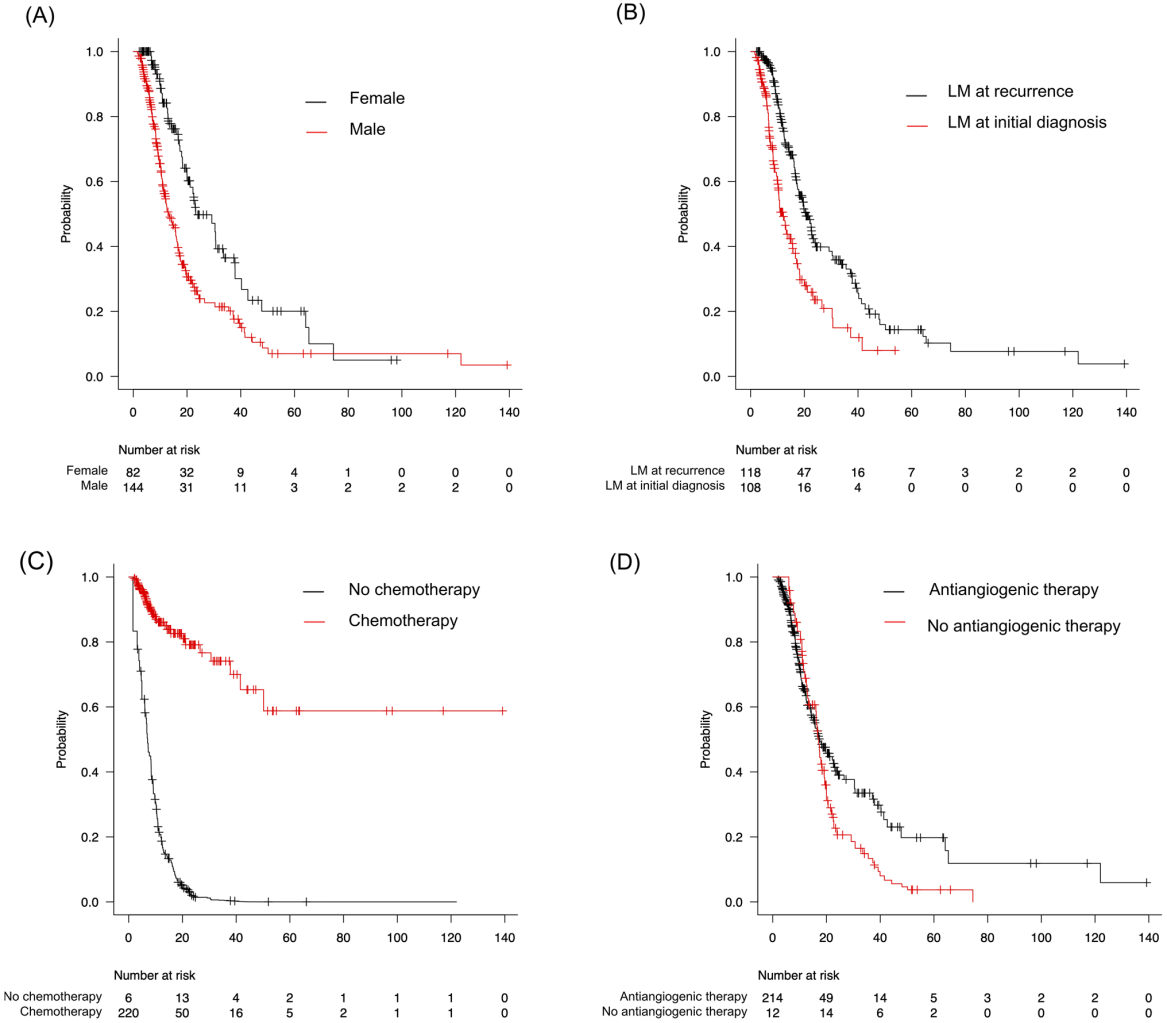
Simon–Makuch curves according to **A** sex, **B** LM at initial diagnosis, **C** chemotherapy, and **D** antiangiogenic therapy in all patients with LM. *LM* leptomeningeal metastases

In all patients, univariable analysis showed that higher KPS (hazard ratio [HR] = 0.95, *P* < 0.001), chemotherapy (HR = 0.07, *P* < 0.001), radiation therapy (HR = 0.12, P < 0.001), and antiangiogenic therapy (HR = 0.34, *P* = 0.002) were predictors of longer OS, while older age (HR = 1.02, *P* < 0.001), male sex (HR = 1.97, *P* < 0.001), histological grade 4 (*P* < 0.001), IDH wildtype (HR = 5.94, *P* < 0.001), MGMT promoter unmethylation (HR = 2.05, *P* < 0.001), and LM at initial diagnosis (HR = 1.97, *P* < 0.001) were predictors of shorter OS. Multivariable analysis revealed that chemotherapy (HR = 0.08, *P* < 0.001) and antiangiogenic therapy (HR = 0.43, *P* < 0.001) were predictors of longer OS, while male sex (HR = 1.48, *P* = 0.038) and LM at initial diagnosis (HR = 1.75, *P* = 0.002) were predictors of shorter OS among patients with LM (Table 2). Simon-Makuch curves according to chemotherapy, antiangiogenic therapy, male sex, and LM at initial diagnosis are shown in Fig. 2.

**Table 2:** Univariable and multivariable time-dependent Cox analyses of glioma patients with LM

Variables	Univariable analysis		Multivariable analysis	
	HR (95% CI)	*P*	HR (95% CI)	*P*
Age at glioma diagnosis	1.02 (1.00–1.04)	< 0.001	1.01 (1.00–1.02)	0.138
Male sex	1.97 (1.41–2.75)	< 0.001	1.48 (1.02–2.13)	0.038
Histological grade 4	3.01 (1.85–4.90)	< 0.001	1.15 (0.64–2.04)	0.638
IDH wildtype	5.94 (2.56–13.79)	< 0.001	2.60 (0.92–7.31)	0.071
1p/19q codeletion	0.49 (0.22–1.13)	0.094		
H3 K27M alteration	1.45 (0.76–2.76)	0.263		
MGMT promoter unmethylation	2.05 (1.41–2.99)	< 0.001	1.32 (0.88–1.99)	0.180
Nonlobar location	1.30 (0.93–1.84)	0.133		
LM at initial diagnosis	1.97 (1.41–2.75)	< 0.001	1.75 (1.22–2.52)	0.002
Disseminated LM	1.14 (0.77–1.67)	0.515		
KPS	0.95 (0.94–0.96)	< 0.001	0.99 (0.98–1.01)	0.213
Gross total resection	0.91 (0.66–1.25)	0.548		
Chemotherapy	0.07 (0.05–0.19)	< 0.001	0.08 (0.05–0.15)	< 0.001
Radiation therapy	0.12 (0.09–1.18)	< 0.001	0.79 (0.46–1.36)	0.400
Antiangiogenic therapy	0.34 (0.17–0.67)	0.002	0.43 (0.28–0.64)	< 0.001
VP shunt insertion	1.18 (0.76–1.83)	0.456		
Experimental therapy	2.14 (0.52–8.82)	0.293		

*CI* confidence interval, *HR* hazard ratio, *IDH* isocitrate dehydrogenase, *LM* leptomeningeal metastases, *MGMT* O^6^-methylguanine-methyltransferase

### Predictors of OS in patients with initial LM

Among patients with initial LM, univariable analysis revealed that higher KPS (HR = 0.95, *P* < 0.001), chemotherapy (HR = 0.17, *P* = 0.001), radiation therapy (HR = 0.09, *P* < 0.001), and antiangiogenic therapy (HR = 0.34, *P* = 0.002) were predictors of longer OS, while older age (HR = 1.03, *P* = 0.008), male sex (HR = 2.07, *P* = 0.015), IDH wildtype (HR = 24.94, *P* = 0.040), and MGMT promoter unmethylation (HR = 1.90, *P* = 0.034) were predictors of shorter OS. Multivariable analysis showed that higher KPS (HR = 0.96, *P* < 0.001), chemotherapy (HR = 0.18, *P* = 0.002), and antiangiogenic therapy (HR = 0.34, *P* = 0.004) were predictors of longer OS, while male sex (HR = 2.17, *P* = 0.012) was a predictor of shorter OS (Table 3). Adjusted and unadjusted Kaplan–Meier curves according to KPS, chemotherapy, antiangiogenic therapy, and male sex are shown in Supplementary Fig. 2.

**Table 3:** Univariable and multivariable Cox analyses of patients with initial LM

Variables	Univariable analysis		Multivariable analysis	
	HR (95% CI)	*P*	HR (95% CI)	*P*
Age at glioma diagnosis	1.03 (1.09–1.05)	0.008	–	–
Male sex	2.07 (1.16–3.71)	0.015	2.17 (1.19–3.97)	0.012
Histological grade 4	2.23 (0.78–6.39)	0.134		
IDH wildtype	24.94 (0.49–1261.94)	0.040	13.21 (5.39–23.43)	0.964
1p/19q codeletion	0.80 (0.20–3.29)	0.759		
H3 K27M alteration	1.35 (0.33–5.56)	0.676		
MGMT promoter unmethylation	1.90 (1.05–3.43)	0.034	–	–
Nonlobar location	1.56 (0.95–2.58)	0.080		
Disseminated LM	0.88 (0.50–1.58)	0.675		
KPS	0.95 (0.93–0.97)	< 0.001	0.96 (0.94–0.98)	< 0.001
Gross total resection	1.20 (0.72–2.02)	0.485		
Chemotherapy	0.17 (0.06–0.50)	0.001	0.18 (0.06–0.55)	0.002
Radiation therapy	0.09 (0.03–0.30)	< 0.001	–	–
Antiangiogenic therapy	0.34 (0.17–0.67)	0.002	0.34 (0.16–0.71)	0.004
VP shunt insertion	1.32 (0.77–2.28)	0.311		
Experimental therapy	0.05 (0.00–795.9)	0.622		

*CI* confidence interval, *HR* hazard ratio, *IDH* isocitrate dehydrogenase, *LM* leptomeningeal metastases, *MGMT* O^6^-methylguanine-methyltransferase

### Predictors of OS in patients with recurrent LM

Among patients with recurrent LM, univariable analysis revealed that longer interval between initial glioma and LM diagnoses (HR = 0.93, *P* < 0.001), gross total resection (HR = 0.60, *P* = 0.025) and chemotherapy (HR = 0.15, *P* = 0.001) were predictors of longer OS, while male sex (HR = 1.83, *P* = 0.009), histological grade 4 (*P* = 0.001), IDH wildtype (HR = 4.32, *P* = 0.001), and MGMT promoter unmethylation (HR = 2.00, *P* = 0.005) were predictors of shorter OS. Multivariable analysis showed that longer interval between initial glioma and LM diagnoses (HR = 0.88, *P* < 0.001) and chemotherapy (HR = 0.04, *P* < 0.001) were predictors of longer OS, while male sex (HR = 1.86, *P* = 0.014) was a predictor of shorter OS (Supplementary Table 1). Adjusted and unadjusted Kaplan–Meier curves according to interval between initial glioma and LM diagnoses, chemotherapy, and male sex are shown in Supplementary Fig. 3.

### Predictors of OS in IDH-wildtype glioblastoma patients with LM

When identical analyses were performed in IDH-wildtype glioblastoma patients (entire patients with LM [n = 179], initial LM [n = 82], and recurrent LM [n = 97]), a similar trend of results to that of entire glioma patients was observed. Supplementary Tables 2–4 show the univariable and multivariable results.

## Discussion

In this study, we comprehensively investigated prognostic factors in adult glioma patients with LM. The median OS of LM in glioma patient was 17.0 months, suggesting that LM may be no longer considered a dismal condition. Considering the increased incidence of LM in glioma patients, along with increasing long-term survivors, determination of strong predictors of survival is crucial for guiding therapeutic decisions. Our findings demonstrated that chemotherapy and antiangiogenic therapy, LM at initial glioma diagnosis, and demographic factors such as sex were independent predictors of LM. Therefore, manifestation of LM in glioma patients should not be considered a terminal condition; instead, active treatments should be considered to extend survival. The female survival advantage suggests that sex may be a relevant factor when planning treatment strategies for patients with LM, whereas longer interval between initial glioma and LM diagnoses should be considered as a more favorable course in patients with recurrent LM.

Previous studies have consistently demonstrated that chemotherapy improves survival of patients with LM [7, 26, 27], which is corroborated in our results. In contrast, radiation therapy was not an independent prognostic factor in our study. Although previous study results have shown that radiation therapy may prolong survival when combined with chemotherapy [28], radiation therapy alone did not have significant effects on survival [26, 27], which could explain our findings. Antiangiogenic therapy has shown discrepant results on survival in patients with LM [5–10]. Our results demonstrating the efficacy of antiangiogenic therapy may be explained based on the underlying pathogenesis. Antiangiogenic therapy inhibits the vascular endothelial growth factor (VEGF), thereby inhibiting co-opting of tumor cells with pre-existing host vessels via VEGF upregulation in the CSF and thus inhibiting LMs [29, 30]. There is need to prospectively assess the role of treatments to validate our results and establish a standardized treatment strategy.

Histological and molecular markers have not been comprehensively examined in previous studies owing to limited data. A recent study in 188 patients with LM showed that survival significantly differed among oligodendroglioma, astrocytoma, and glioblastoma [7]; however, diagnosis was performed only histopathologically without molecular markers. Our Kaplan–Meier results showed that survival of patients with LM differed according to molecular types (log-rank *P* < 0.001). However, contrary to our initial expectation, aggressive molecular and histological features did not remain as significant prognostic factors on multivariable analyses. Because majority of LM patients in our dataset already had IDH wildtype, MGMT unmethylation, or histological grade 4 (94.8%, 68.6% and 85.8%, respectively), these markers may not have remained as statistically significant prognostic factors on multivariable results. Our previous study showed that patients with IDH wildtype, MGMT unmethylation, or histological grade 4 were more likely to develop LM [3], which explains the high proportion of aggressive molecular and histological markers in LM.

Demographic and clinical factors such as sex, initial LM, and KPS were independent predictors of LM. In previous studies of glioma patients with LM in smaller datasets, demographic factors such as age or sex were not reported as prognostic factors [7, 26, 27, 31, 32], while one study reported KPS as a significant factor in 34 patients [27]. Previously patients with glioblastoma have demonstrated sexually dimorphic patterns, with males exhibiting poor OS than females [33, 34]. Interestingly, this female survival advantage remained in glioma patients with LM, suggesting a sex-specific mechanism persistently affecting the survival of patients with LM. Longer interval between glioma and recurrent LM diagnoses as a favorable prognostic factor in recurrent LM may be explained by the fact that gliomas developing LM at a later course may suggest an underlying indolent biological activity.

This study had some limitations. First, the study analyzed a single-center, retrospective dataset; thus, there was inevitable heterogeneity in diagnosis and treatment over the long study period. Second, CSF cytology or flow cytometry was performed for a small proportion of patients diagnosed with LM [35, 36]. Because of its invasiveness and low diagnostic value [37], CSF cytology is not routinely performed in clinics [36]; the current NCCN guideline suggests that LM should be diagnosed in the presence of positive radiologic findings with supportive clinical findings [38]. Third, biologically distinct types of gliomas were included in a single group for analysis, which may limit the interpretation of our study.

In conclusion, active treatment with chemotherapy and antiangiogenic therapy may result in survival benefits for patients with glioma with LM. There is consistent female survival advantage in both patients with initial and recurrent LMs, whereas longer interval between glioma and LM development suggests longer OS in recurrent LM.

## Supplementary Information

Below is the link to the electronic supplementary material.

Supplementary file1 (DOCX 44 kb)

Supplementary file2 (TIF 1453 kb)

Supplementary file3 (TIF 3828 kb)

Supplementary file4 (TIF 2377 kb)

## Abbreviations

CSF: Cerebrospinal fluid
IDH: Isocitrate dehydrogenase
KPS: Karnofsky performance status
LM: Leptomeningeal metastases
MGMT: O^6^-methylguanine-DNA methyltransferase
OS: Overall survival
WHO: World Health Organization
