# Supplementary material for: Revisiting prognostic factors in glioma with leptomeningeal metastases: a comprehensive analysis of clinical and molecular factors and treatment modalities
Source: J Neurooncol. 2023 Feb 25;162(1):59–68. doi: 10.1007/s11060-022-04233-y (PMC10050057; doi:10.1007/s11060-022-04233-y)
Supplement: Supplementary file 1 — Supplementary file1 (DOCX 44 kb) [file 11060_2022_4233_MOESM1_ESM.docx]

**S1. MRI Protocol**

A 3T MRI unit (Achieva or Ingenia; Philips Healthcare) and an 8-channel sensitivity encoding head coil were used for brain MRI. The protocol included T1-weighted turbo spin-echo images with inversion recovery (repetition time [TR], 2000 ms; echo time [TE], 10 ms; inversion time [TI], 1000 ms; field of view [FOV], 240 mm; section thickness, 5 mm; matrix, 256 × 256), T2-weighted turbo spin-echo (TR, 3000 ms; TE, 80 ms; FOV, 240 mm; section thickness, 5 mm; matrix, 256 × 256), and T2-weighted fluid-attenuated inversion recovery (FLAIR) (TR, 10,000 ms; TE, 125 ms; TI, 2500 ms; FOV, 240 mm; section thickness, 5 mm; matrix, 256 × 256) images. 3D T1-weighted turbo field echo images (TR, 9.8 ms; TE, 4.6 ms; FOV, 240 mm; section thickness, 1 mm; matrix, 224 × 224) were acquired after the injection of gadolinium-based contrast (0.1 mL/kg of gadobutrol, Gadovist; Bayer Schering Pharma, Berlin, Germany). Post-contrast FLAIR (TR, 10,000-11,000 ms; TE, 110-125 ms; TI, 2500 ms; FOV, 230-240 mm; section thickness, 5 mm; matrix, 256 × 256) was subsequently acquired 10 minutes and 30 seconds after the injection of gadolinium-based contrast.

Spine MRI was obtained using a 1.5T unit (Achieva dStream; Philips Healthcare). The protocol included sagittal T1-weighted (TR, 450 ms; TE, 9.8 ms; section thickness, 3 mm; matrix: 314 × 448; FOV, 350 mm), axial T1-weighted (TR, 520 ms; TE, 9.3 ms; section thickness, 3 mm; matrix, 202 x 384; FOV, 250 mm), axial T2-weighted (TR, 3430 ms; TE, 120 ms; section thickness, 3 mm; matrix, 202 x 384; FOV, 250 mm), and sagittal T2-weighted (TR, 3760 ms; TE, 100 ms; section thickness, 3 mm; matrix, 338 × 512; FOV, 350 mm) images. Contrast-enhanced T1WIs in axial and sagittal planes were acquired after the injection of gadolinium-based contrast (0.1 mL/kg of gadobutrol, Gadovist; Bayer Schering Pharma, Berlin, Germany).

**S2. Diagnosis of LM**

Cases detected by the initial database inquiry and MRI review were manually confirmed by a chart review, including clinical notes and pathology reports. For confirmation of presence or absence of LM, all MRI data from patients were reviewed independently from the MRI report. LM was diagnosed in patients whose brain or spine MRI reviews showed LM or with positive CSF cytology based on the pathology report.

On MRI, brain LM was defined as linear or nodular leptomeningeal enhancement or linear ependymal enhancement, cranial nerve root enhancement; spine LM was defined as linear or nodular leptomeningeal enhancement or spinal nerve root enhancement.^2,3^ True leptomeningeal enhancement on post-contrast FLAIR could be confirmed by using the pre-contrast FLAIR as the reference image; abnormalities on pre-contrast FLAIR without additional enhancement on post-contrast FLAIR were not diagnosed as LM, according to the Response Assessment in Neuro-Oncology (RANO) recommendation ^2^. For confirmatory diagnosis of equivocal cases, only patients with follow-up MRIs demonstrating consistent leptomeningeal enhancement were diagnosed as LM. Disseminated LM and subependymal LM were separately recorded according to the previous criteria.^1^ Disseminated LM was defined as leptomeningeal or nerve root enhancement, whereas subependymal LM was defined as a subependymal or ependymal enhancement on MRI, which is radiographically indistinguishable. Imaging diagnosis was established with a two-week washout period after imaging feature evaluation by independent review of two neuroradiologists (with 11 and 18 years of experience, respectively). In the rare case of ambiguity, a senior neuroradiologist (with 30 years of experience) was consulted for the final decision.

**Supplementary Figure Legends.**

**Supplementary Figure 1.** Kaplan-Meier curves according to the types of glioma in the entire LM patients.

LM = leptomeningeal metastases

**Supplementary Figure 2**. Adjusted and unadjusted Kaplan-Meier curves according to (A) sex, (B) KPS (< 70), (C) chemotherapy, and (D) antiangiogenic therapy in patients with initial LM.

KPS = Karnofsky performance status; LM = leptomeningeal metastases

**Supplementary Figure 3**. Adjusted and unadjusted Kaplan-Meier curves according to (A) interval between initial glioma and LM diagnoses, (B) sex, and (C) chemotherapy in patients with recurrent LM.

LM = leptomeningeal metastases

**Supplementary Table 1.** Univariable and multivariable Cox analyses of patients with recurrent LM

| Variables | Univariable analysis | | Multivariable analysis | |
| --- | --- | --- | --- | --- |
|  | HR (95% CI) | *P* | HR (95% CI) | *P* |
| Age at glioma diagnosis | 1.02 (1.00–1.03) | 0.084 |  |  |
| Interval between initial glioma and LM diagnoses (months) | 0.93 (0.91-0.95) | <0.001 | 0.88 (0.85–0.91) | <0.001 |
| Male sex | 1.83 (1.16–2.90) | 0.009 | 1.86 (1.14–3.05) | 0.014 |
| Histological grade 4 | 2.60 (1.50–4.49) | 0.001 | – | – |
| IDH wildtype | 4.32 (1.82–10.28) | 0.001 | 2.47 (0.88-6.98) | 0.088 |
| 1p/19q codeletion | 0.50 (0.18–1.40) | 0.189 |  |  |
| H3 K27M alteration | 1.71 (0.78–3.75) | 0.182 |  |  |
| MGMT promoter unmethylation | 2.00 (1.23–3.27) | 0.005 | – | – |
| Nonlobar location | 1.11 (0.69–1.80) | 0.670 |  |  |
| Disseminated LM | 1.34 (0.80–2.26) | 0.270 |  |  |
| KPS | 0.98 (0.97–1.00) | 0.076 |  |  |
| Gross total resection | 0.60 (0.39–0.94) | 0.025 | – | – |
| Chemotherapy | 0.15 (0.05–0.47) | 0.001 | 0.04 (0.10–0.18) | <0.001 |
| Radiation therapy | 1.12 (0.62–2.03) | 0.705 |  |  |
| Antiangiogenic therapy | 0.78 (0.50–1.20) | 0.253 |  |  |
| VP shunt insertion | 1.00 (0.53–1.90) | 0.991 |  |  |
| Experimental therapy | 1.15 (0.36–3.65) | 0.814 |  |  |

CI, confidence interval; HR, hazard ratio; IDH, isocitrate dehydrogenase; LM, leptomeningeal metastases; MGMT, O^6^-methylguanine-methyltransferase

**Supplementary Table 2.** Univariable and multivariable time-dependent Cox analyses of IDH-wildtype glioblastoma patients with LM

| Variables | Univariable analysis | | Multivariable analysis | |
| --- | --- | --- | --- | --- |
|  | HR (95% CI) | *P* | HR (95% CI) | *P* |
| Age at glioma diagnosis | 1.02 (1.00–1.03) | 0.046 | 1.02 (1.01-1.03) | 0.009 |
| Male sex | 2.05 (1.39–3.02) | <0.001 | 1.66 (1.11-2.48) | 0.013 |
| MGMT promoter unmethylation | 1.66 (1.10–2.49) | 0.015 | 1.39 (0.99-2.15) | 0.135 |
| Nonlobar location | 1.27 (0.89–1.82) | 0.200 |  |  |
| LM at initial diagnosis | 1.76 (1.24–2.49) | 0.001 | 1.76 (1.22-2.53) | 0.002 |
| Disseminated LM | 0.93 (0.61–1.43) | 0.751 |  |  |
| Gross total resection | 1.29 (0.92–1.81) | 0.146 |  |  |
| KPS | 0.952 (0.94–0.963) | <0.001 | 0.9 (0.97-1.00) | 0.055 |
| Chemotherapy | 0.09 (0.06–0.14) | <0.001 | 0.10 (0.06-0.18) | <0.001 |
| Radiation therapy | 0.16 (0.11–0.24) | <0.001 | 1.18 (0.69-2.03) | 0.542 |
| Antiangiogenic therapy | 1.05 (0.71–1.54) | 0.809 |  |  |
| VP shunt insertion | 1.04 (0.65–1.64) | 0.880 |  |  |
| Experimental therapy | 1.64 (0.39–6.87) | 0.498 |  |  |

CI, confidence interval; HR, hazard ratio; LM, leptomeningeal metastases; MGMT, O^6^-methylguanine-methyltransferase

**Supplementary Table 3.** Univariable and multivariable Cox analyses of IDH-wildtype glioblastoma patients with initial LM

| Variables | Univariable analysis | | Multivariable analysis | |
| --- | --- | --- | --- | --- |
|  | HR (95% CI) | *P* | HR (95% CI) | *P* |
| Age at glioma diagnosis | 1.03 (1.00–1.05) | 0.018 | – | – |
| Male sex | 2.09 (1.14–3.82) | 0.017 | 1.83 (1.23-3.20) | 0.009 |
| MGMT promoter unmethylation | 1.54 (0.82–2.90) | 0.181 |  |  |
| Nonlobar location | 1.41 (0.83–2.39) | 0.199 |  |  |
| Disseminated LM | 1.01 (0.56–1.85) | 0.965 |  |  |
| Gross total resection | 1.41 (0.83–2.38) | 0.208 |  |  |
| KPS | 0.96 (0.94–0.98) | <0.001 | 0.96 (0.94–0.98) | 0.001 |
| Chemotherapy | 0.18 (0.06–0.53) | 0.002 | 0.41 (0.20–0.85) | 0.002 |
| Radiation therapy | 0.09 (0.03–0.31) | <0.001 | – | – |
| Antiangiogenic therapy | 0.34 (0.17–0.69) | 0.003 | 0.41 (0.20–0.85) | 0.017 |
| VP shunt insertion | 1.08 (0.62–1.89) | 0.784 |  |  |

CI, confidence interval; LM, leptomeningeal metastases; MGMT, O^6^-methylguanine-methyltransferase; HR, hazard ratio

**Supplementary Table 4.** Univariable and multivariable Cox analyses of IDH-wildtype glioblastoma patients with recurrent LM

| Variables | Univariable analysis | | Multivariable analysis | |
| --- | --- | --- | --- | --- |
|  | HR (95% CI) | *P* | HR (95% CI) | *P* |
| Age at glioma diagnosis | 1.00 (0.98–1.02) | 0.829 |  |  |
| Interval between initial glioma and LM diagnoses (months) | 0.91 (0.89–0.94) | <0.001 | 0.81 (0.77–0.86) | <0.001 |
| Male sex | 1.98 (1.18–3.30) | 0.009 | 2.147(1.24–3.80) | 0.006 |
| MGMT promoter unmethylation | 1.67 (0.97–2.86) | 0.062 |  |  |
| Nonlobar location | 1.15 (0.69–1.93) | 0.583 |  |  |
| Disseminated LM | 0.96 (0.52–1.75) | 0.885 |  |  |
| Gross total resection | 0.94 (0.58–1.54) | 0.812 |  |  |
| KPS | 1.00 (0.98–1.02) | 0.928 |  |  |
| Chemotherapy | 0.18 (0.04–0.72) | 0.016 | 0.01 (0.00–0.02) | <0.001 |
| Radiation therapy | 1.19 (0.65–2.17) | 0.581 |  |  |
| Antiangiogenic therapy | 0.44 (0.28–0.71) | 0.001 | 0.31 (0.18–0.54) | <0.001 |
| VP shunt insertion | 10.76 (0.39–1.48) | 0.413 |  |  |
| Experimental therapy | 0.57 (0.14–2.32) | 0.428 |  |  |

CI, confidence interval; LM, leptomeningeal metastases; MGMT, O^6^-methylguanine-methyltransferase; HR, hazard ratio.

**References**

1. Andersen BM, Miranda C, Hatzoglou V, DeAngelis LM, Miller AM: Leptomeningeal metastases in glioma: The Memorial Sloan Kettering Cancer Center experience. **Neurology 92:**e2483-e2491, 2019

2. Chamberlain M, Junck L, Brandsma D, Soffietti R, Rudà R, Raizer J, et al: Leptomeningeal metastases: a RANO proposal for response criteria. **Neuro Oncol 19:**484-492, 2017

3. Le Rhun E, Weller M, Brandsma D, Van den Bent M, de Azambuja E, Henriksson R, et al: EANO-ESMO Clinical Practice Guidelines for diagnosis, treatment and follow-up of patients with leptomeningeal metastasis from solid tumours. **Ann Oncol 28:**iv84-iv99, 2017
