# Supplementary figures and images for: Revisiting prognostic factors in glioma with leptomeningeal metastases: a comprehensive analysis of clinical and molecular factors and treatment modalities
Source: J Neurooncol. 2023 Feb 25;162(1):59–68. doi: 10.1007/s11060-022-04233-y (PMC10050057; doi:10.1007/s11060-022-04233-y)

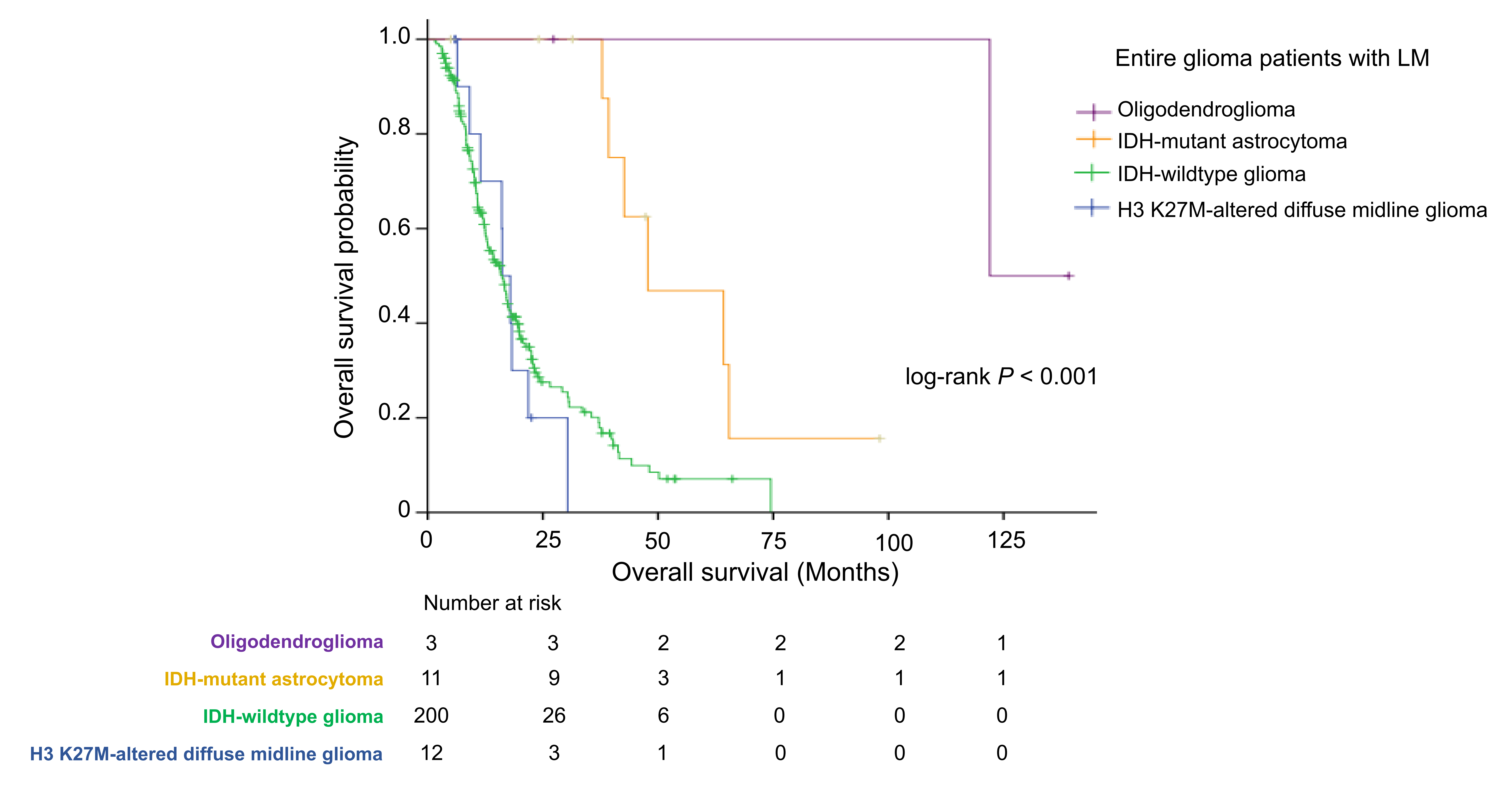

Supplement: Supplementary file 2 — Supplementary file2 (TIF 1453 kb) [file 11060_2022_4233_MOESM2_ESM.tif]

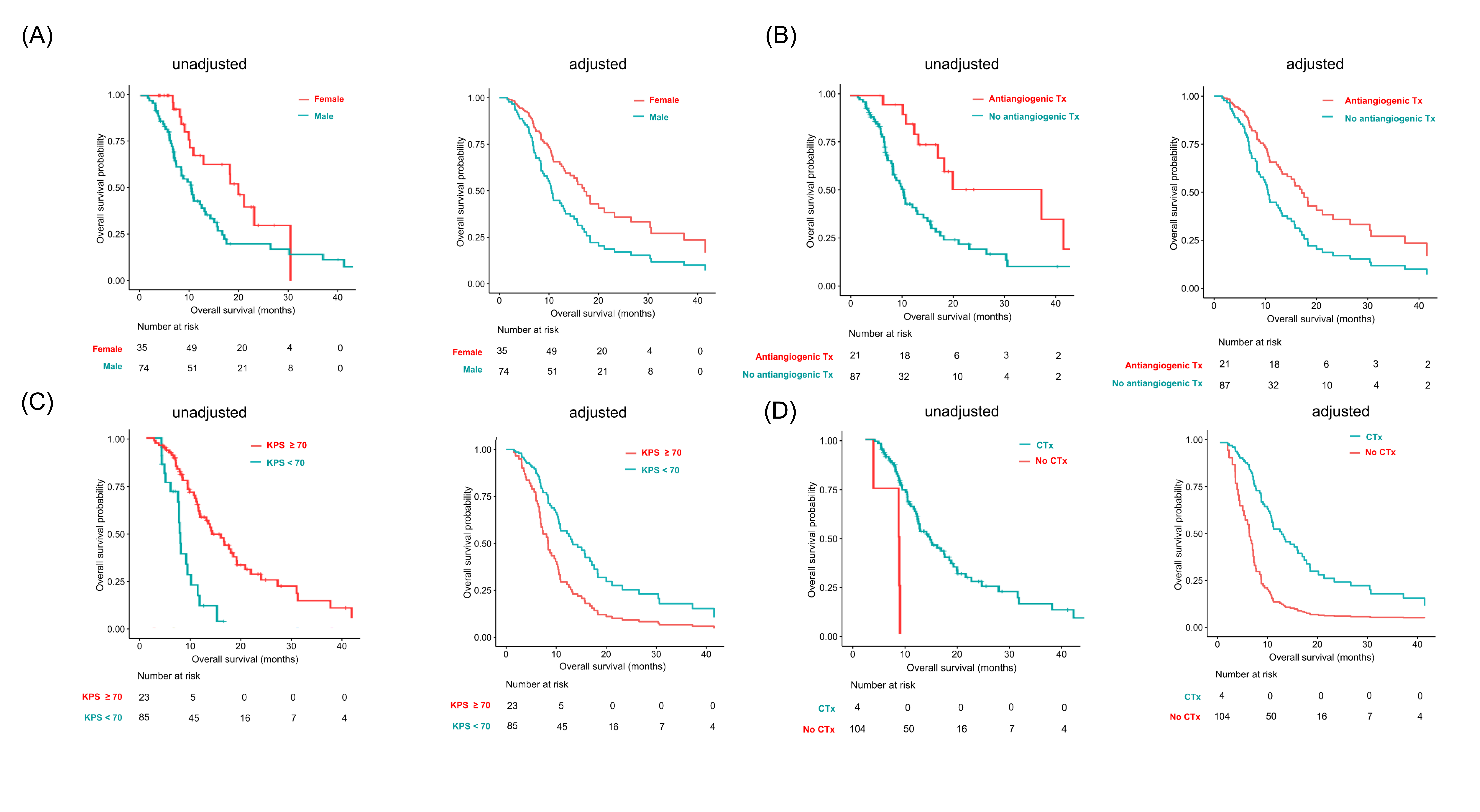

Supplement: Supplementary file 3 — Supplementary file3 (TIF 3828 kb) [file 11060_2022_4233_MOESM3_ESM.tif]

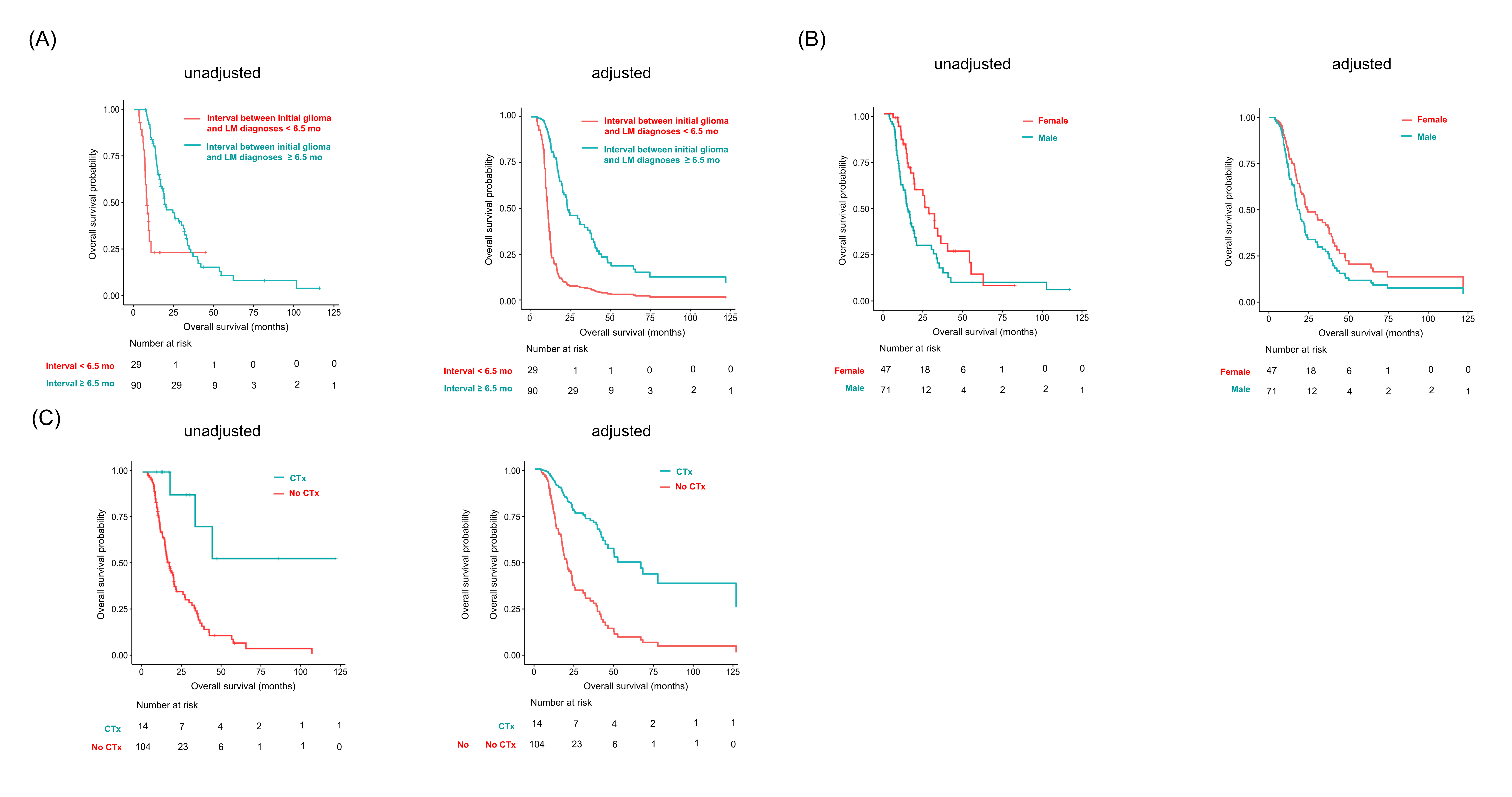

Supplement: Supplementary file 4 — Supplementary file4 (TIF 2377 kb) [file 11060_2022_4233_MOESM4_ESM.tif]
